# Notes of the Week

**Published:** 1921-08-20

**Authors:** 


### NOTES OF THE WEEK.

### GRANTS FROM THE VOLUNTARY HOSPITALS COMMISSION.

We are officially informed that the Voluntary Hospitals Commission continue to receive numerous applications from individual hospitals. To avoid misunderstanding, they are anxious to make it known that grants will only be made on the recommendation in London of King Edward's Fund, or in the provinces of the local Voluntary Hospitals Committees. Steps are now being taken in co-operation with the County and County Borough Councils to establish these local Committees, and any inquiry as to whether a Committee has already been appointed for a particular area should be addressed to the Clerk of the County Council. Hospitals are asked to defer their applications until the local Hospital Committee has been appointed; and in no case should any hospital apply direct to the Commission.

### THE "SUSSEX" SCHEME.

The organisers of the National Provident Scheme for Hospital and Additional Medical Service have been very shy of making public any details of the plan on which they propose to work when the three London hospitals (the London, St. Thomas's, and the Royal Free) which have adopted the scheme put it into force as from October 1. They have had a conference with representatives of some of the Friendly and Approved Societies, but nothing has been allowed to transpire except that a resolution was carried unanimously expressing the opinion that the services made available by the National Provident Scheme are likely to prove of great value to members of friendly and approved societies, and the conference recommended the several committees of management and their officers to bring to the notice of their members the opportunity which is given them by the organising committee of joining the scheme. But in order to arouse the necessary interest in the scheme we would suggest that a much more active Press campaign should be instituted and that the public as well as the profession and the hospitals should be supplied with fuller information on many points than they have at present.

### HIGHER CHARGES FOR DISTANT PATIENTS?

The Queen's Hospital, Birmingham, is now making a charge of three guineas a week on in-patients, able to pay, who live beyond the radius of Greater Birmingham. This has, perhaps not unnaturally, created a good deal of comment, and the house governor, Mr. Hedley Lucas, has had to explain to the Solihull Guardians, and, indeed, to many patients living at the distance named, that inability to pay will be considered in their case as in that of those nearer to the hospital. Mr. Lucas has added that not more than a total of ten guineas will in any event be payable for a patient's whole stay, but how many patients stay more than three weeks? Is not the average length of stay shorter? We ask because the argument sounds as if the Committee felt that the charge was too high and was anxious to make it appear lower. At present it is not clear whether the policy is intended to discourage patients from a distance by charging them a higher sum, because if the higher sum is not enforced it will not act as a deterrent. Such an idea, however, would be interesting to discuss, and, as misconception exists concerning the intention and the practice at the Queen's Hospital, Mr. Hedley Lucas should welcome an opportunity to explain the position.

### THE LOWESTOFT EXPERIMENT.

The Committee of the Lowestoft and North Suffolk Hospital has decided to adopt a contributory scheme which, if successful, may do away with the necessity to impose any scale of patients' payments. The employers in each trade will be invited to form their own Hospital Aid Committee, appoint their own secretary, and pay the money collected into the bank on the plan which has succeeded well at Ipswich. Ward committees, presumably in charge of collections in the different parts of the town, are also to be formed to approach those whom the Aid Committee does not touch. We have now sufficient experience to show that there is no part of the country where schemes of this character cannot be introduced successfully.

### A NEW MODEL INSTITUTION.

The Shropshire Orthopædic Hospital, which was begun at Baschurch by Miss Hunt, was opened at Oswestry by the Marchioness of Cambridge. It accommodates some 200 children and 100 pensioners, and has attracted patients and visitors from distant counties and many parts of the world. The hospital was formerly a military hospital for Park Hall Camp. Sir Robert Jones, the Liverpool surgeon, described it at the opening as the first of open-air hospitals. In America, which he has just left, two or three hospitals have been built upon its model. There need not be cripples, he added. If the State would help, one need hardly ever see a pair of crutches.

### TRAGIC DEATH OF DR. CUFF.

We deeply regret to record the tragic death of Dr. Herbert Cuff, principal medical officer to the Metropolitan Asylums Board, who was drowned on August 15 whilst trying to rescue his two young daughters from drowning on the coast of Norfolk. The two girls, aged fourteen and twelve, were bathing with a girl friend, when a strong tide carried them out to sea. Dr Cuff witnessed the incident, and went to their help; but although all were capable swimmers, the friend alone reached safety, and the doctor as well as the two children were drowned. Dr. Cuff was resident head of the Belgian Refugee Camp at the Alexandra Palace during the war, for which he was decorated with the O.B.E.

He had been head of the North Eastern Fever Hospital under the M.A.B. before becoming principal medical officer to that body, and had written well-known text-books for nurses. We offer to Mrs. Cuff our deep sympathy in the tragic bereavement which has overtaken her.

### THE RUSSIAN FAMINE.

The Joint Council of the British Red Cross Society and the Order of St. John are supporting the appeal which has been issued by the Imperial War Relief Fund to help the victims of the famine in Russia. In order that there shall be no overlapping it is not making a separate appeal, but its chairman (Sir Arthur Stanley) and vice-chairman appeal to the British Red Cross workers throughout the land to make the need of help widely and promptly known and to stir their countrymen to a generous effort. Amongst the signatories to the appeal of the Imperial War Fund, which is working with the Comité International de la Croix Rouge and the International League of Red Cross Societies in Geneva to organise relief, are the Archbishop of Canterbury, the Lord Chancellor (Lord Birkenhead), Lord Crewe, Mrs. Fawcett, and others, who point out that the appeals from Russia are unparalleled in their urgency, and that in the presence of such misery and want no humane man, whatever may be his opinion of recent events in Russia, will allow his instincts of sympathy to be silenced. Subscriptions should be made payable to the honorary treasurer of the Imperial War Relief Fund, earmarked for "Russian Famine Relief," and addressed to the Fund at Fishmongers' Hall, London Bridge, E.C.

### ANCOATS HOSPITAL.

A comparative statement of the first six months' work at Ancoats Hospital, Manchester, has been issued, showing the income and expenditure for the first half of 1919, 1920, and 1921. The numbers of patients attended in all departments have appreciably increased, and the expenditure under several important headings, notably provisions and surgery and dispensary, has fallen from 15 to 20 per cent. If it had not been that salaries and wages and establishment charges have still a tendency to rise, the gross expenditure would have been much lower instead of, as is the case, remaining about the same. The income for the six months has risen from £8,542 to £10,442. Subscriptions are £350 and donations £1,000 higher, and there is every indication that the neighbourhood is appreciative of the work of the hospital.

### INFIRMARY PATIENTS AND MEDICAL ATTENTION.

Wandsworth Board of Guardians have considered the question of the attention given by the medical staff to the patients individually at St. James's Infirmary. Dr. McCormac, the medical superintendent, informed the Committee that he does not recommend any further additions to the present resident medical staff, which consists of five whole-time and two part-time medical officers. There are 750 beds; the average daily number occupied is 620. Generally speaking, there is a systematic examination of each patient once weekly, but many cases of acute illness require and receive frequent and, it may be, lengthy treatment daily. Many of the patients suffering from chronic or minor ailments do not require daily attention from the doctor, and no good would result were the doctors' time so spent. He suggested in order to relieve the staff the appointment of an anæsthetist, and the board decided to accept his proposal, a visiting anæsthetist to be appointed to attend four mornings a week at a fee of 100 guineas a year. The board also agreed that each patient should be examined by a doctor once every week, as it was maintained that this had not been done hitherto.

### GIFTS IN KIND FOR A NURSES' HOME?

The London Jewish Hospital, at Stepney Green, is asking for gifts of furniture to help with the equipment of the new Nurses' Home. It is suggested that many of those who read the appeal possess furniture "suitable for this purpose." We do not wish to discourage this effort, but can a nurses' home, where space, dustlessness, and comfort have to be so carefully adjusted in the relatively small bedrooms, be satisfactorily equipped with odds and ends of furniture? Would it not be better to sell the gifts, and to have the furniture made for the purpose and place it will have to fill? To furnish nurses' bedrooms with stray pieces is not a wise policy, though their sitting-rooms might possibly benefit from it.

### ANTIDOTES TO POISONS.

Much interest has been aroused in medical and pharmaceutical circles regarding the Bill introduced in the House of Commons providing that the receptacle of each poison sold by retail shall bear a printed description of an antidote for that poison. At first sight there seems little objection to such a measure, though there may be a danger, in some instances, of careless people mistaking the poisons so labelled for the antidote, or a mistake may be made through parts of such a label becoming obliterated, leaving the name of the antidote only on the container. We understand that the representative medical and pharmaceutical bodies have not been consulted by the framer of the Bill, and their views are awaited with interest. We believe that cases of poisoning could be lessened materially if more stringent regulations were imposed on the sale of commonly used poisons, such as spirits of salt (hydrochloric acid), etc., which at present can be bought at any oil store without any record of the transaction being filed. Apart from its use in special trades, such as plumbing, etc., its place as a domestic cleansing agent could well be taken by other efficient non-poisonous preparations. It would be of interest to know whether any hospital at present adopts the practice of labelling poisonous preparations, showing the particular antidote for use where required.

